# Obstructive sleep apnea risk and sleep quality in adolescents with polycystic ovary syndrome: a case–control study

**DOI:** 10.1590/1806-9282.20241615

**Published:** 2025-06-02

**Authors:** Sakine Yılmaz, Zekiye Küpçü

**Affiliations:** 1 Karatekin University, Faculty of Health Sciences, Department of Midwifery – Çankırı, Turkey.; 2 Gaziosmanpaşa Education and Research Hospital, Department of Pediatric Endocrinology – İstanbul, Turkey.

**Keywords:** Adolescent, Female, Obstructive, Polycystic ovary syndrome, Sleep apnea, Sleep quality

## Abstract

**OBJECTIVE:**

The aim of this study was to evaluate the risk of obstructive sleep apnea and sleep quality in adolescents with and without polycystic ovary syndrome.

**METHODS:**

A case–control study was conducted on 82 adolescent girls admitted to the pediatric endocrinology clinic of a training and research hospital in Turkey. Data were collected using the Participant Information Form, Stop and Stop-Bang Questionnaire, Cleveland Adolescent Sleepiness Questionnaire, Sleep Quality Scale, and Sleep Variables Questionnaire.

**RESULTS:**

It was determined that the mean Stop and Stop-Bang scores of adolescents in the healthy and polycystic ovary syndrome groups were similar and had a high level of obstructive sleep apnea risk. Daytime sleepiness levels were similar in both groups, but daytime sleepiness mean scores were a little higher in healthy adolescents. In addition, it was determined that the level of sleepiness at school and during transportation was statistically significant and high in the healthy group, and the sleep quality of all adolescents was moderately similar.

**CONCLUSION:**

Our data reveal the importance of a comprehensive assessment of sleep health, including obstructive sleep apnea, as well as duration, timing, and quality in adolescents when considering polycystic ovary syndrome. Healthcare professionals should consider the sleep health of all adolescents presenting to endocrine outpatient clinics.

## INTRODUCTION

Polycystic ovary syndrome (PCOS) is a common endocrine disorder characterized by ovulatory dysfunction and hyperandrogenism that affects 3.39–11.04% of adolescent girls^1,2^. PCOS's common clinical manifestations include menstrual irregularities, hirsutism, dermatologic complaints, such as acne, obesity, impaired quality of life, and depressive and anxious moods^3–5^.

Improving lifestyle management in adolescents is critical to preventing the clinical manifestations of PCOS^1^. It is a risk factor for poor sleep health in adolescents, including sleep duration; timing; quality; obstructive sleep apnea (OSA); and hormonal, metabolic, and environmental factors^1,6^.

OSA is a chronic sleep disorder characterized by intermittent hypoxia and recurrent complete (apnea) or partial (hypopnea) upper airway obstructions during sleep^7^. The prevalence of sleep-disordered breathing in adolescents with PCOS is estimated to be between 0 and 57%^1^. Those with PCOS lack progesterone and estrogen, which are protective in the regulation of respiration during sleep, and have excessive androgens that trigger central adiposity, which increases the risk of OSA by 5–10 times^3,6^. Symptoms of sleep disorders such as difficulty in starting sleep, restless sleep, severe daytime fatigue, and insomnia may be observed in patients with PCOS. Therefore, OSA and sleep quality evaluation are important^6,8^.

Sleep quality is as important as its duration for health and optimal body functioning. Good sleep quality and adequate sleep duration improve an individual's physical performance as well as mental performance. Insufficient sleep or poor quality sleep can lead to decreased academic success and school performance in individuals and various accidents and injuries resulting from carelessness. All these problems particularly affect adolescents^9^.

As a result, sleep disorders and OSA have been reported to worsen insulin resistance, energy balance, presentation of PCOS, and metabolic outcomes in individuals with PCOS^8^. Therefore, evaluating OSA risk, sleep quality, and sleep-related variables in adolescents is important. This study was conducted to evaluate the risk of OSA and sleep quality in adolescents with and without a diagnosis of PCOS.

The research questions are as follows:

- Is there a difference in the risk of OSA in adolescents with and without PCOS?
- Is there a difference in sleep quality between these two groups?

## METHODS

### Ethical considerations

Written approval was obtained from the university ethics board (2023/03-09). Adolescents younger than 18 years of age gave consent and were informed, and written and verbal informed consents were obtained from their parents. This research was conducted following the Helsinki Declaration.

### Study design and participants

The research was conducted as a case–control study. The study was applied to the pediatric endocrinology outpatient clinic of a training and research hospital between February 06 and May 31, 2023. The study sample consisted of females of the same ages (between the ages of 10 and 19 years) allocated to two groups, the PCOS group and the control group. Inclusion criteria for the PCOS group: being a female patient diagnosed with PCOS according to the Rotterdam criteria, being literate, and participating in the study voluntarily. According to the Rotterdam Consensus, PCOS is defined by the presence of two of three of the following criteria: oligo-anovulation, hyperandrogenism, and polycystic ovaries (≥12 follicles measuring 2–9 mm in diameter and/or an ovarian volume >10 mL in at least one ovary)^10–12^. Inclusion criteria for the control group: being a female patient who was not diagnosed with PCOS, being literate, and voluntarily participating in the study. Using the data of a sample study^13^, this study was completed with 82 adolescents with a 95% confidence interval (1-α) and d=0.746 effect size, and the power of the test (1-β) was determined as 91.5% as a result of post hoc power.

### Data collection tools

The data were collected using the "Participant Information Form," developed by the researchers based on the relevant literature, "Stop and Stop-Bang Questionnaire" (STOP-BANG), "Cleveland Adolescent Sleepiness Questionnaire," and "Sleep Quality Scale" and "Sleep Variables Questionnaire" (SQS-SVQ).

The Participant Information Form consists of seven questions to determine sociodemographic (age, height, and weight) and menstrual cycle characteristics (pattern, age, and duration)^3,6,14^.

The STOP-BANG consists of eight items related to the clinical features of sleep apnea, and the questions are of yes/no type. The total score is between 0 and 8. In the STOP-BANG questionnaire, if the answer to three out of the eight questions is yes, it is considered high risk. STOP-BANG score ≥3 is classified as moderate and >15 as severe^15,16^.

In the Cleveland Adolescent Sleepiness Questionnaire, 11 items are scored positively and 5 items (2, 5, 7, 11, and 13) are scored negatively. Daytime sleepiness score is obtained by summing the scores of the 16 items. The total score ranges from 16 to 80, with higher scores indicating higher levels of daytime sleepiness. The scale consists of four sub-dimensions. The Cronbach's alpha value of the scale is found to be 0.89^17^. In our study, the Cronbach's alpha value of the Cleveland Adolescent Sleepiness Questionnaire was 0.87.

The SQS-SVQ consists of seven scale items and eight survey items to assess sleep quality. The scale consists of factors such as sleep quality (1, 2, 3, 4, 5, 6, and 7), parental control (8), time spent in bed on school days (9 and 13), and total sleep time (9, 13, 14, and 15). In the scale, items 1, 2, 3, 4, and 7 are reversed to calculate the total score obtained from the seven items; 7 points indicate poor sleep quality and 21 points indicate good sleep quality. The Cronbach's alpha coefficient of the scale is 0.72^9^. In our study, the Cronbach's alpha value of the scale was 0.66.

### Data analysis

SPSS statistical package (version 23.0) was used to analyze the data. Number, percentage, mean, median, and standard deviation values were used for descriptive statistics. Compliance with normal distribution was analyzed using Shapiro-Wilk and Kolmogorov-Smirnov tests. Yates correction and chi-square test were used to compare categorical variables according to groups. Mann-Whitney U test was used to compare non-normally distributed data, and the independent-samples t-test was used to compare normally distributed data. Spearman's rho correlation coefficient was used to correlate non-normally distributed data, and Pearson's correlation coefficient was used to correlate normally distributed data. The statistical significance level was accepted as p<0.05.

## RESULTS

The mean body mass index (BMI) of the PCOS group (25.4±5.1 kg/m^2^) was statistically significantly higher than that of the control group (21.8±4.0 kg/m^2^) (p=0.001). There was a statistically significant difference in groups regarding mean BMI standard deviation score (SDS) (p=0.001). There was a statistically significant difference in the menstrual pattern and duration of menstruation in the PCOS and control groups (p<0.05; Table 1).

**Table 1:** Comparison of demographic characteristics between control and polycystic ovary syndrome groups (n=82).

Sociodemographic characteristics	Control group (n=39)		PCOS group (n=43)		Total (n=82)		Test statistic	p*
	Mean±SD	Median (min–max)	Mean±SD	Median (min–max)	Mean±SD	Median (min–max)		
Age	15.3±1.5	15.5 (12.8–17.4)	15.5±1.5	15.6 (11.9–17.8)	15.40±1.50	15.56 (11.93–17.80)	764.50	0.492
BMI	21.8±4.0	21.4 (15.1–32.5)	25.4±5.1	24.2 (17.4–37.8)	23.72±4.94	22.05 (15.08–37.83)	485.50	0.001
BMI SDS	0.0±1.6	0.1 (-4.5 to 3.0)	1.2±1.4	1.2 (-2.0 to 3.7)	0.64±1.62	0.44 (-4.53 to 3.67)	495.00	0.001
Age at menarche	12.5±1.1	12.0 (11.0–15.0)	12.1±1.5	12.0 (9.0–15.5)	12.27±1.32	12.00 (9.00–15.50)	703.50	0.200
	Control group (n=39)		PCOS group (n=43)	Total (n=82)		Test statistic		p
Duration of menstruation								
Less than 3 days	0 (0.0)		1 (2.3)	1 (1.2)		6.487		0.039^1^
3–8 days	38 (97.4)^a^		34 (79.1)^b^	72 (87.8)				
More than 8 days	1 (2.6)^a^		8 (18.6)^b^	9 (11.0)				

BMI: body mass index; SDS: standard deviation score; PCOS: polycystic ovary syndrome; Mean±SD: mean±standard deviation; Min–max: minimum–maximum.

* Mann-Whitney U test.

1 Yates correction.

a and b There is no difference between groups with the same letter within each row.

Statistically significant values are denoted in bold.

There was a statistically significant difference between the mean scores of sleepiness at school according to the groups: 11.5±4.8 in the control group and 9.1±4.80 in the PCOS group (p=0.010). There was a statistically significant difference between the mean sleepiness scores during transportation: 7.5±3.3 in the control group and 6.0±3.4 in the PCOS group (p=0.018). The mean total score of sleep duration in the control group was 416.9±111.2 min, while the mean score of the group with PCOS was 420.7±91.6 min (p=0.964) (Table 2).

**Table 2:** Scale score distributions between control and polycystic ovary syndrome groups (n=82).

	Control group (n=39)		PCOS group (n=43)		Test statistic	p
	Mean±SD	Median (min–max)	Mean±SD	Median (min–max)		
Stop and Stop-Bang Score	3.9±0.8	4.0 (3.0–6.0)	4.2±0.7	4.0 (3.0–6.0)	680.000^1^	0.109
Cleveland Adolescent Daytime Sleepiness Total score	44.7±9.8	44.0 (27.0–66.0)	41.0±11.5	41.0 (22.0–72.0)	1.559^2^	0.123
Sleepiness at school score	11.5±4.8	12.0 (5.0–22.0)	9.1±4.80	7.0 (5.0–23.0)	564.500^1^	**0.010**
Insomnia at school score	16.1±3.6	17.0 (6.0–22.0)	17.0±4.5	18.0 (6.0–25.0)	-0.902^2^	0.370
Evening sleepiness score	9.7±2.8	9.0 (3.0–15.0)	9.0±3.3	9.0 (3.0–15.0)	0.970^2^	0.335
Sleepiness during transportation score	7.5±3.3	7.0 (3.0–14.0)	6.0±3.4	5.0 (3.0–14.0)	585.500^1^	**0.018**
Sleep quality total score	13.7±2.5	14.0 (8.0–18.0)	13.6±2.4	13.0 (8.0–18.0)	0.248^2^	0.805
Total sleep duration score	416.9±111.2	415.0 (210.0–718.0)	420.7±91.6	405.0 (210.0–695.0)	756.000^1^	0.964
Sleep efficiency score	87.2±11.5	90.0 (43.8–99.7)	86.6±9.6	87.2 (58.3–99.4)	699.500^1^	0.542

1 Mann-Whitney U test.

2 Independent-sample t-test.

SD: standard deviation; PCOS: polycystic ovary syndrome. Statistically significant values are denoted in bold.

In the control group, there was a statistically significant positive moderate relationship between age and the Cleveland Adolescent Daytime Sleepiness Total score (r=0.404; p=0.011) and school sleepiness score (r=0.437; p=0.005). There was a statistically significant positive weak relationship between age and sleepiness during transportation (r=0.359; p=0.025). There was a statistically significant negative relationship between age and total sleep duration (r=-0.422; p=0.007). There was a statistically significant negative weak relationship between age and sleep efficiency (r=-0.361; p=0.024). There was a statistically significant negative weak relationship between BMI and the Cleveland Adolescent Daytime Sleepiness Total score (r=-0.326; p=0.043). There was a statistically significant negative, weak relationship between BMI SDS and the Cleveland Adolescent Daytime Sleepiness Total score (r=-0.376; p=0.018). There was a statistically significant negative relationship between BMI SDS and sleepiness at school (r=-0.350; p=0.029). There was a statistically significant positive moderate relationship between age at menarche and insomnia at school (r=0.492; p=0.00; Table 3).

**Table 3:** Examination of the relationship between some sociodemographic characteristics and scale scores between groups.

Groups		Age		BMI		BMI SDS		Age of menarche	
		r*	p	r*	p	r*	p	r*	p
Control group	Stop and Stop-Bang Score	0.010	0.950	0.165	0.316	0.168	0.308	-0.076	0.647
	Cleveland Adolescent Daytime Sleepiness Total score	0.404	**0.011**	-0.326	**0.043**	-0.376	**0.018**	0.251	0.124
	Sleepiness at school score	0.437	**0.005**	-0.301	0.063	-0.350	**0.029**	0.076	0.647
	Insomnia at school score	0.101	0.542	-0.200	0.221	-0.226	0.167	0.492	**0.001**
	Evening sleepiness score	0.298	0.066	-0.260	0.110	-0.309	0.055	0.062	0.706
	Sleepiness during transportation score	0.359	**0.025**	-0.004	0.980	-0.021	0.899	0.017	0.916
	Sleep quality total score	0.079	0.632	-0.048	0.773	-0.076	0.647	0.133	0.419
	Total sleep duration score	-0.422	**0.007**	-0.036	0.829	0.029	0.860	-0.173	0.292
	Sleep efficiency score	-0.361	**0.024**	0.015	0.926	0.067	0.687	-0.071	0.668
PCOS group	Stop and Stop-Bang Score	-0.043	0.783	0.381	**0.012**	0.390	**0.010**	-0.058	0.712
	Cleveland Adolescent Daytime Sleepiness Total score	0.045	0.773	-0.056	0.722	-0.097**	0.536	-0.224**	0.150
	Sleepiness at school	0.211	0.174	-0.081	0.604	-0.094	0.548	-0.090	0.565
	Insomnia at school	0.015	0.925	-0.114	0.466	-0.189**	0.224	-0.165**	0.289
	Evening sleepiness	-0.165	0.291	0.040	0.799	0.044**	0.781	-0.267**	0.084
	Sleepiness during transportation	0.018	0.910	-0.052	0.739	-0.050	0.752	-0.068	0.663
	Sleep quality total score	-0.082	0.602	-0.065	0.677	-0.013**	0.933	0.134**	0.390
	Total sleep duration	-0.087	0.598	0.027	0.871	0.023	0.890	-0.163	0.321
	Sleep efficiency	-0.110	0.506	-0.074	0.653	-0.068	0.679	-0.209	0.201

* Spearman's rho correlation coefficient,

** Pearson's correlation coefficient. BMI: body mass index; SDS: standard deviation score; PCOS: polycystic ovary syndrome. Statistically significant values are denoted in bold.

There was a statistically significant positive weak correlation between BMI and the STOP-BANG score in the PCOS group (r=0.381; p=0.012). There was a statistically significant positive weak relationship between BMI SDS and the STOP-BANG score (r=0.39; p=0.010, Table 3).

## DISCUSSION

PCOS is a common endocrine disease in adolescents. PCOS may include features such as central adiposity, hyperandrogenism, low progesterone, and estradiol levels, all of which play a role in the pathophysiology of OSA^11,18^. PCOS in adults is associated with sleep disorders and OSA^3,6,19,20^. However, little is known about these associations in adolescents.

In this study, it was determined that adolescents in the healthy and PCOS groups had similar mean STOP-BANG scores and had a high risk of OSA. In the literature, studies conducted on adolescents^21^ and young people^22^ found that the risk of OSA was high. Simon et al. found that sleep-disordered breathing was significantly worse in adolescents with PCOS^1^. Predisposing factors defined for OSA include obesity, age, smoking, and craniofacial and upper airway morphologic abnormalities^23^. In a meta-analysis study, the collective prevalence of OSA was found to be higher in adults than in adolescents^3^. Although our study result is similar to the literature, it is thought that the fact that all the adolescents had an average BMI above 21 and more than half of them were diagnosed with PCOS may have influenced the high risk of OSA.

Our study determined that daytime sleepiness levels were similar in both groups. In addition, the statistically significant and high level of sleepiness at school and during transportation in the healthy group in our study is a very striking result. In contrast to our study, Simon et al. reported that adolescents with obesity and PCOS had worse sleep, compared with those with obesity without PCOS^24^. Another study found that adolescent girls with PCOS treated with metformin (850 mg twice daily) showed significant improvements in self-reported sleep disturbances and daytime sleepiness and BMI^25^. It is thought that the difference in our results from the literature may be due to the characteristics of the population, the fact that neither group had any other chronic disease, and the fact that the adolescents with PCOS did not receive any treatment for the disease.

It has been suggested that obesity and hormonal factors caused by PCOS show an impaired sleep quality^6,20^. This study determined that the sleep quality of adolescents in the healthy and PCOS groups was moderately similar. In studies conducted on adult women, sleep quality, time to fall asleep, use of sleep medication, and daytime dysfunction were reported to be worse in the PCOS group^26,27^. In a study conducted on women with PCOS, including adolescent girls, sleep quality was found to be poor^28^. No other study demonstrating sleep quality in adolescents with PCOS was found in the literature. Our study result is very important in terms of literature findings. The fact that adolescence is a period of physical, sexual, physiological, and social changes for girls and that this process is perceived differently and experienced in different ways by each adolescent may have affected our results. In addition, it is thought that the difference in our results from the literature may also be due to the different educational, income, and socio-cultural conditions of the families of adolescents.

## CONCLUSION

In this study, healthy and PCOS adolescents had a high risk of OSA. The statistically significant and high level of sleepiness found in the healthy group at school and during transportation is a remarkable result. The sleep quality of healthy and PCOS adolescents is moderate. Our data reveal the importance of a comprehensive assessment of sleep health, including OSA, as well as duration, timing, and quality in adolescents when considering PCOS. Healthcare professionals (nurses, doctors, and midwives) should consider the sleep health of all adolescents presenting to endocrine outpatient clinics.
